# Evaluation of national institute for health and care excellence guidance for ruptured abdominal aortic aneurysms by emulating a hypothetical target trial

**DOI:** 10.3389/fcvm.2023.1219744

**Published:** 2023-07-28

**Authors:** Wolf Eilenberg, Mohammed A. Waduud, Henry Davies, Marc A. Bailey, D. Julian A. Scott, Florian Wolf, Anna Sotir, Sebastian Lakowitsch, Alexandra Kaider, Georg Heinze, Christine Brostjan, Christoph M. Domenig, Christoph Neumayer

**Affiliations:** ^1^ Division of Vascular Surgery, Department of General Surgery, Medical University of Vienna, Vienna, Austria; ^2^ Leeds Institute of Cardiovascular and Metabolic Medicine, University of Leeds, Leeds, United Kingdom; ^3^ Leeds Vascular Institute, Leeds General Infirmary, Leeds, United Kingdom; ^4^ Division of Cardiovascular and Interventional Radiology, Department of Biomedical Imaging and Image Guided Therapy, Medical University of Vienna, Vienna, Austria; ^5^ Section for Clinical Biometrics, Center for Medical Data Science, Medical University of Vienna, Vienna, Austria

**Keywords:** aortic aneurysm, infrarenal, ruptured, open repair, endovascular aneurysm repair, guidelines

## Abstract

**Objective:**

This retrospective study evaluates the performance of UK National Institute for Health and Care Excellence (NICE) Guidelines on management of ruptured abdominal aortic aneurysms in a “real world setting” by emulating a hypothetical target trial with data from two European Aortic Centers.

**Methods:**

Clinical data was retrospectively collected for all patients who had undergone ruptured endovascular aneurysm repair (rEVAR) and ruptured open surgical repair (rOSR). Survival analysis was performed comparing NICE compliance to usual care strategy. NICE compliers were defined as: female patients undergoing rEVAR; male patients >70 years old undergoing rEVAR; and male patients ≤70 years old undergoing rOSR. Hemodynamic instability was considered additionally.

**Results:**

This multicenter study included 298 patients treated for rAAA. The majority of patients were treated with rOSR (186 rOSR vs. 112 rEVAR). Overall, 184 deaths (68 [37%] with rEVAR and 116 [63%] with rOSR) were observed during the study period. Overall survival under usual care was 69.2% at 30 days, 56.5% at one year, and 42.4% at 5 years. NICE compliance gave survival outcomes of 73.1% at 30 days, 60.2% at 1 year and 42.9% at 5 years. The risk ratios at these time points, comparing NICE-compliance to usual care, were 0.88, 0.92 and 0.99, respectively.

**Conclusions:**

We support NICE recommendations to manage men below the age of 71 years and hemodynamic stability with rOSR. There was a slight survival advantage for NICE compliers overall, in men >70 years and women of all ages.

## Introduction

Ruptured endovascular aneurysm repair (rEVAR) is a minimally invasive treatment modality for patients presenting with a ruptured abdominal aortic aneurysm (rAAA). It is associated with reduced post-operative morbidity and mortality (1–6). The National Institute for Health and Care Excellence (NICE) guidelines, in the United Kingdom (UK) advocate the use of rEVAR in this elderly, often multi-morbid, patient population. The guidelines suggest that rEVAR should be considered in men above the age of 70 years and all women with suitable anatomy (7). However, in practice decisions about management of rAAA are influenced by local expertise, service availability and organizational arrangements, AAA anatomy and hemodynamic status at admission. We sought to interrogate a retrospective series of rAAA from two European aortic centers to determine if stratification of patients based on compliance with UK NICE Guidelines for rAAA (hemodynamic stability, age and gender criteria) compared to our usual care would uncover a difference in overall survival. Since patients were not randomized between usual care and stratification based on NICE guidelines, we used novel methodology to emulate a hypothetical target trial from our observational data.

## Material and methods

### Study population

The study population consisted of patients treated for a ruptured infra-renal AAA in two aortic centers (Leeds, UK [Center 1] and Vienna, Austria [Center 2]). Patients in Center 1 were identified between 2007 and 2016, and in Center 2 between 2000 and 2016. All patients were treated prior to the publication of the first draft of the current NICE guidelines. Patients with ruptured iliac aneurysms, juxtarenal aortic aneurysms, thoraco-abdominal aortic aneurysms, mycotic aneurysms and ruptured abdominal-iliac aneurysm were excluded from the study.

The stability of the patients was defined according to the main clinical definition of blood pressure of at least 80 mmHg mean systolic (8). Treatment decisions were determined based on: anatomical criteria for rEVAR according the device instruction for use and hemodynamic stability of the patient on arrival at the hospital. EVAR teams were available 24/7, 365 days a year in both centers across the duration of the study. EVAR teams were called in once it was known that there was a patient on route, or the decision has been taken to re-offer rEVAR. rEVAR was performed via femoral access in all cases. The decision to perform rEVAR with a bifurcated graft or aorto-uni-iliac configuration in conjunction with a femoro- femoral bypass was left to the discretion of the operating team. rOSR was performed in most of the cases using either a mid-line or vertical approach depending on operator preference, after achieving proximal aortic control at either the supracoeliac, supra-renal or infra-renal aortic level, the rOSR was performed by using either a tube graft or a bifurcated graft. Grafts made of knitted polyester (Dacron) or polytetrafluoroethylene (PTFE) grafts were used at the discretion of the operating surgeon. Permissive hypotension and low volume fluid administration was permitted as standard (9).

### Data collection and variables for analysis

Patients were identified from registries of rAAA performed at each center. The following demographic variables and comorbidities, recorded at time of surgery were included in the analysis: age, gender, body mass index (BMI), history of smoking (yes/no), hypertension (HTN), diabetes mellitus, hypercholesterolemia, coronary artery disease (CAD), cerebrovascular disease, pulmonary disease and any known malignancy. Renal function was assessed using glomerular filtration rate (GFR) and creatinine. Patient hemodynamic stability at presentation was determined from electronic case records. The date and cause of death was identified from case records. Overall survival was calculated as the number of days from surgery to death from any cause or last follow-up date. whatever occurred earlier. Survival time was treated as censored if a patient was alive at last follow-up.

The study was approved by the local ethics review committee (EC 2086/2012) at Center 2, conducted according to the Declaration of Helsinki and registered the study ID research registry 5,637. According to the local authorities at Center 1, ethics committee approval was not deemed necessary for this retrospective study. The study adhered to the STROCSS criteria (10). Given that all procedures were performed under emergency conditions it was not possible to obtain a complete history in all patients and missing values are reported in Table 1.

**Table 1:** Patient demographic characteristics by intervention.

Characteristic		Leeds (Center 1)		Vienna (Center 2)		*P*-value
		rEVAR (*n* = 58)	rOSR (*n* = 88)	rEVAR (*n* = 58)	rOSR (*n* = 98)	
Age (years)	Mean (SD)	76.5 (10.3)	**7**6.8 (6.9)	75.7 (8.2)	69.8 (10.2)	<.0001^a^
Male	*n*/*n*_observed_ (%)	48/58 (82.8%)	72/88 (81.8%)	45/54 (83.3%)	84/98 (85.7%)	.533^b^
BMI (kg/m^2^)	Mean (SD)*	26.9 (4.4)	28.6 (5.2)	27.1 (4.5)	28.1 (5.3)	.864^a^
Smoking history	*n*/*n*_observed_* (%)	34/57 (59.6%)	45/82 (54.9%)	14/42 (33.3%)	29/86 (33.7%)	.0001^b^
Co-morbidities						
– Hypertension	*n*/*n*_observed_* (%)	14/58 (24.1%)	31/88 (35.2%)	39/53 (73.6%)	55/95 (57.9%)	<.0001^b^
– Diabetes mellitus	*n*/*n*_observed_* (%)	4/58 (6.9%)	12/88 (13.6%)	13/54 (24.1%)	15/95 (15.8%)	.059^b^
– Dyslipidaemia	*n*/*n*_observed_* (%)	33/58 (56.9%)	53/88 (60.2%)	16/22 (72.7%)	22/66 (33.3%)	.020^b^
– Coronary heart disease	*n*/*n*_observed_* (%)	20/58 (34.5%)	24/88 (27.3%)	27/53 (50.9%)	35/92 (38.0%)	.025^b^
– Cerebrovascular disease	*n*/*n*_observed_* (%)	2/58 (3.5%)	3/88 (3.4%)	12/53 (22.6%)	21/91 (23.1%)	<.0001^b^
– Chronic lung disease	*n*/*n*_observed_* (%)	7/58 (12.1%)	15/88 (17.1%)	19/48 (39.6%)	29/94 (30.9%)	.0002^b^
– Malignancy	*n*/*n*_observed_* (%)	8/58 (13.8%)	2/88 (2.3%)	9/20 (45.0%)	7/91 (87.7%)	.046^b^
– Creatinine (mg/dl)	Median (IQR)*	1.0 (0.9–1.3)	1.1 (0.9–1.5)	1.3 (1.1–1.6)	1.1 (1.0–1.4)	.004^c^
– eGFR (ml/min)	Mean (SD)*	60.3 (18.7)	57.6 (20.6)	54.3 (24.1)	71.4 (31.1)	.025^a^
– Stability	*n*/*n*_observed_* (%)	28/41 (68.3%)	40/59 (67.8%)	42/53 (79.3%)	46/98 (46.9%)	.120^b^
– Chronic kidney disease (eGFR<60 ml/min)	*n*/*n*_observed_* (%)	26/58 (44.8%)	41/88 (46.6%)	35/52 (67.3%)	35/98 (35.7%)	.894^b^

* Frequency of missing values: *n* = 75 (BMI)/*n* = 31 (Smoking history)/*n* = 4 (Hypertension)/*n* = 3 (Diabetes Mellitus)/*n* = 64 (Dyslipidaemia)/*n* = 7 (Coronary heart disease)/*n* = 8 (Cerebrovascular disease)/*n* = 10 (Chronic lung disease)/*n* = 41 (Malignancy)/*n* = 3 (Creatinine)/*n* = 20 (eGFR)/*n* = 47 (Stability)/*n* = 2 (Chronic Kidney Disease).

^a^ *T*-test.

^b^ Chi–Square test.

^c^ Wilcoxon rank sum test.

### Definition of compliers of NICE criteria and modified NICE criteria

Female patients, male patients older than 70 years with rEVAR, and male patients up to 70 years of age with rOSR were considered as NICE-compliers. Female patients, and male patients older than 70 years with rOSR, and male patients up to 70 years of age with rEVAR were considered as NICE-non-compliers. To additionally account for the patients' hemodynamic stability, we defined the following modified NICE (mNICE) criteria: Hemodynamically unstable patients treated with rOSR, and stable patients treated according to the NICE criteria were considered as mNICE-compliers. On the other hand, hemodynamically unstable patients treated with rEVAR, and stable patients treated contradictory to the NICE criteria were considered as mNICE-non-compliers.

### Statistical methods

Categorical variables are described by absolute numbers and relative frequencies, and continuous variables by mean and standard deviation (SD) or median and interquartile range (IQR) in case of non-normal distribution. The inverse Kaplan–Meier method (11) was used to estimate the median follow-up time. To evaluate the impact of the proposed NICE Guidelines in rAAA patients on overall survival, we emulated a hypothetical target trial comparing the outcome under usual care to that expected under compliance to NICE (12). The hypothetical target trial methodology simulates a randomized trial in which patients are randomized to either usual care or treatment according to NICE criteria. The outcomes under both alternatives are then statistically compared. The outcome under usual care can be estimated from our total study cohort, and the sub-cohort of NICE-compliers serves as the basis to estimate the outcome if all patients were treated compliant to NICE. However, these patients are a sub-cohort of the total study cohort and thus might differ in their baseline characteristics from the total cohort. Therefore, we first evaluated differences in baseline characteristics between the total cohort and the NICE-compliers by propensity score (multivariable logistic regression) analyses using compliance to NICE (yes/no) as binary outcome variable and age, sex, diabetes mellitus, chronic lung disease, chronic kidney disease, and stability as covariates. The inverted predicted probabilities of compliance (inverse probability of treatment weights, IPW) were then used to weight the outcomes in the sub-cohort of NICE-compliers (13). Considering patients of center 1 (*n* = 146), the variable stability was not included in the propensity score model, since it was missing in 32% of the cases. On the other hand, 14 observations were excluded from the propensity score model of center 2, due to missing values in the baseline variables diabetes, chronic lung disease, chronic kidney disease, and/or stability, resulting in a total sample size of *n* = 138 patients in center 2. Altogether, *n* = 284 patients were used for statistical comparisons of overall survival. In a second step, using the unweighted observations of the total cohort or the IPW-weighted observations of the NICE-compliers, the Kaplan–Meier method was applied to estimate survival probabilities, and risk ratios were calculated to compare the two cohorts at the pre-specified time points 30 days, 1 year, and 5 years after procedure. This Kaplan–Meier analysis was also performed for the subgroups of men aged ≤70 years, of men aged >70 years, and of women, respectively. Bootstrap percentile confidence intervals were calculated for the risk ratios, using the balanced bootstrap method with 10,000 samples stratified by the center. This method was used to account for the special dependency-structure of the two cohorts (the “NICE-complier” cohort is an IPW-weighted sub-cohort of the “usual care” cohort). To evaluate the impact of the modified NICE criteria compared to NICE and usual care, only patients of center 2 were considered (*n* = 138). Patients of center 1 were excluded from this sub-analysis due to the large number of missing values in the baseline variable stability. Again, propensity score models were used and the three IPW-weighted cohorts (“mNICE compliers”, “NICE compliers”, and “usual care”) were compared with respect to overall survival as described before. All statistical analyses were performed using the software system SAS (version 9.4. (2016), SAS Institute Inc. Cary, NC, USA).

## Results

### Patient characteristics

**Figure 1:**
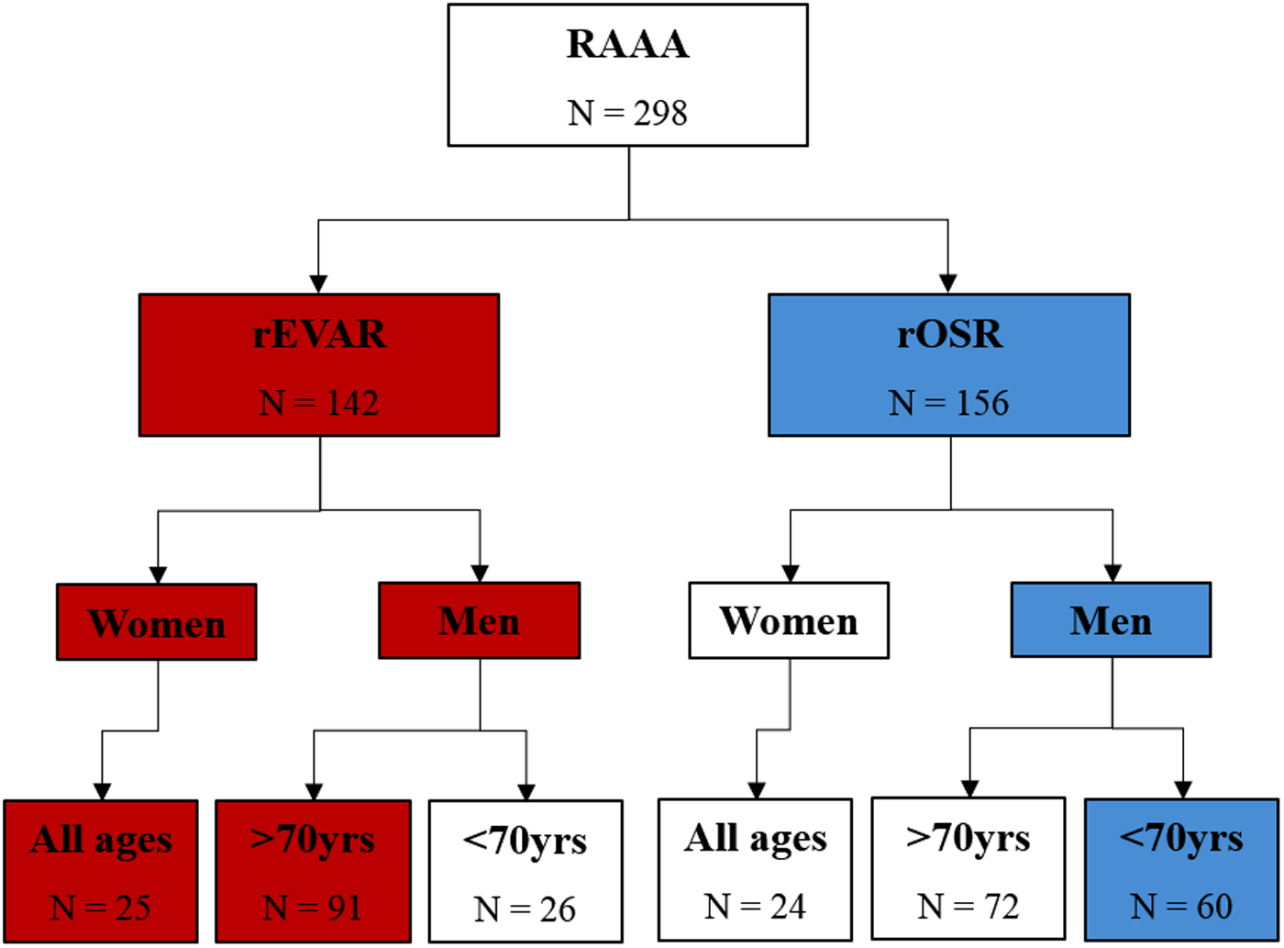
Flow diagram of study population. Red indicates patients who met current recommended criteria for ruptured endovascular aortic repair (rEVAR). Blue indicates patients who met current recommended criteria for ruptured open surgical repair (rOSR).

This study included 298 patients with a rAAA, 112 EVARs were performed (58 patients from Center 1 and 54 patients from Center 2) and 186 OSRs were performed (88 patients from Center 1 and 98 patients from Centre 2), Figure 1.

The mean (SD) age of patients included in the study was 74.3 (9.5) years and 249 patients (83.6%) were men. Baseline characteristics are summarized in Table 1 and Supplementary Table S1. The median (IQR) follow-up time was 5.9 (4.6–7.2) and 7.4 (1.8–10.1) years in Center 1 and 2, respectively. Morphological characteristics of rAAA could be assessed for 102 patients from Center 1 and for 104 patients from Center 2. The data was extrapolated from preoperative computer tomography (CT) scans and is depicted in Table 2.

**Table 2:** Morphological characteristics of rAAA by intervention.

Characteristic		Leeds (Center 1)		*P*-value	Vienna (Center 2)		*P*-value
		rEVAR (*n* = 51)	rOSR (*n* = 51)		rEVAR (*n* = 54)	rOSR (*n* = 50)	
Aortic neck diameter (mm)	Median (IQR)	23 (21–27)	23 (20–26)	.765	25 (21–30)	21 (0–28)	.041
Aortic neck length (mm)	Median (IQR)	22 (15–30)	6 (0–20)	<.001	22 (15–26)	25 (0–39)	.934
Aneurysm of EIA							
Left EIA	*n*/*n*_observed_ (%)	11/51 (21.6%)	6/51 (11.8%)	.184	2/54 (3.7%)	1/50 (2%)	.604
Right EIA	*n*/*n*_observed_ (%)	8/51 (15.7%)	5/51 (9.8%)	.373	5/54 (9.3%)	4/50 (8%)	.819
Right and left EIA	*n*/*n*_observed_ (%)	6/51 (11.8%)	16/51 (31.4%)	.016	28/54 (51.9%)	12/50 (24%)	.004

Data are presented as *n*/%), median (interquartile range). EIA, external iliac aneurysm.

### Evaluation of NICE guidelines

Examining how treatment of the patients relates to the NICE recommendations, there were 144 NICE compliers (19 women undergoing rEVAR; 66 men >70 years undergoing rEVAR; and 59 men ≤70 years undergoing rOSR), representing 48.3% of the study population. There were 154 patients not compliant with NICE recommendations (30 women undergoing rOSR; 27 men ≤70 years undergoing rEVAR; and 97 men >70 years undergoing rOSR, representing 51.7% of the study population, Figure 1.

### Mortality following rAAA, cumulative survival and risk ratios

**Figure 2:**
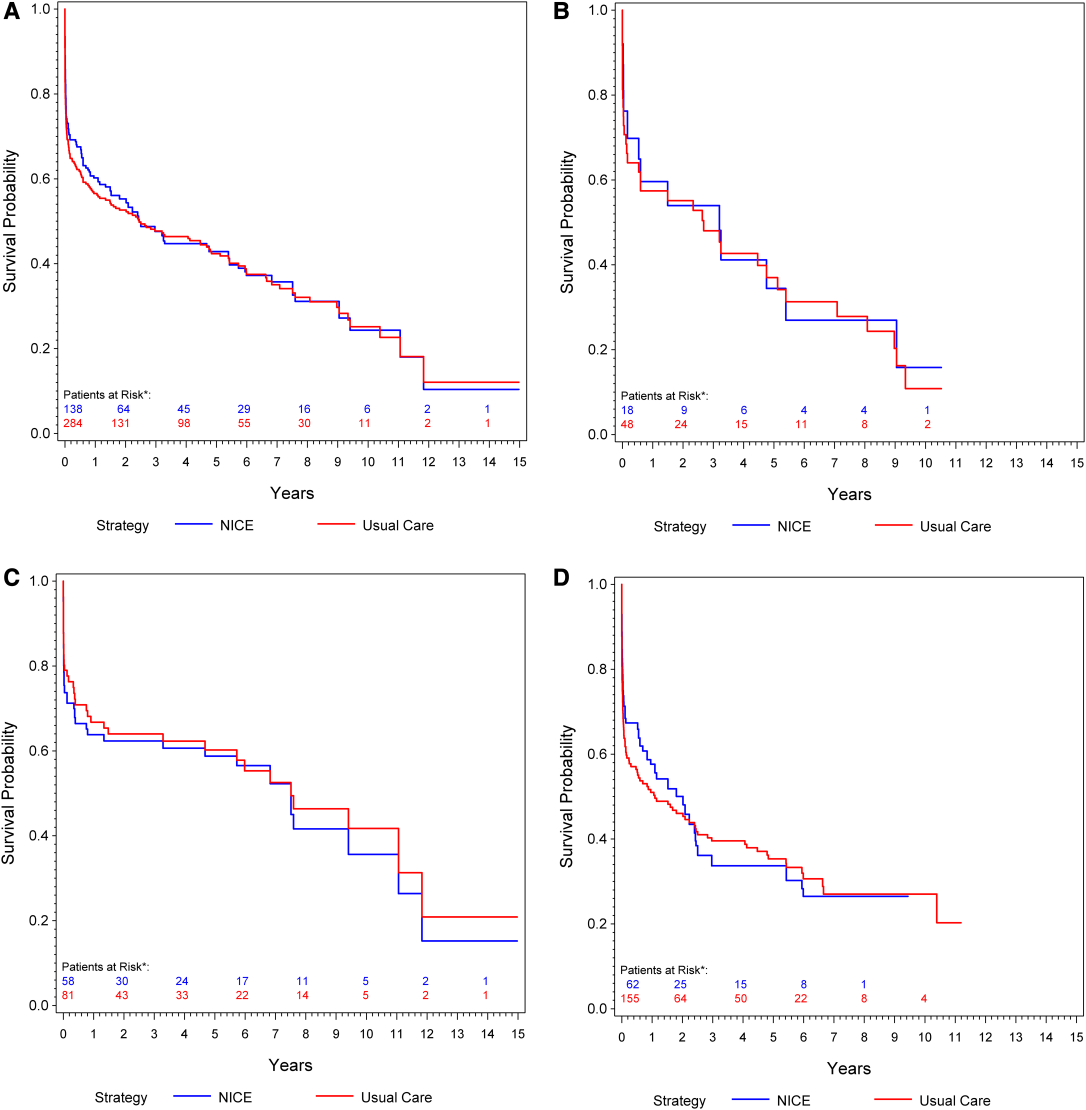
(**A**) Kaplan–meier curve: cumulative survival of patients undergoing rOSR and rEVAR according to the NICE criteria compared to the usual care cohort. (**B**) Kaplan–Meier curve of female patients: cumulative survival of patients undergoing rOSR and rEVAR according to the NICE criteria compared to the usual care cohort. (**C**) Kaplan–Meier curve of men aged ≤70 years: cumulative survival of patients undergoing rOSR and rEVAR according to the NICE criteria compared to the usual care cohort. (**D**) Kaplan–Meier curve of men aged >70 years: cumulative survival of patients undergoing rOSR and rEVAR according to the NICE criteria compared to the usual care cohort. *The actually observed numbers of patients at risk in the NICE-complier cohort, and the NICE-modified-complier cohort, respectively, are given. However, IPW-weighted observations were used for calculation of the Kaplan-Meier curve.

Overall, 184 deaths (68 [37%] with rEVAR and 116 [63%] with rOSR) were observed during the study period. The IPW-weighted Kaplan–Meier estimate of cumulative survival under compliance to NICE is compared to the unweighted estimate under usual care for the total cohort is shown in Figure 2A. Overall survival under usual care is estimated as 69.2% at 30 days, 56.5% at one year, and 42.4% at 5 years. Under compliance to NICE the corresponding numbers were 73.1%, 60.2% and 42.9%, respectively Figure 2A. The risk ratios at these time points, comparing NICE-compliance to usual care, were 0.88 [95% Confidence-Interval: 0.67–1.09], 0.92 [0.76–1.08], and 0.99 [0.87–1.12], respectively Table 3.

**Table 3:** Risk ratios (RR) of mortality at 30 days, 1 year and 5 years: overall, in men aged ≤70 years, in men aged above 70 years and in women of all ages.

	Overall all-cause mortality		
	30 days RR [95% BCI*]	1 year RR [95% BCI*]	5 years RR [95% BCI*]
Overall: NICE-compliers vs. usual care	0.88 [0.67–1.09]	0.92 [0.76–1.08]	0.99 [0.87–1.12]
Men aged ≤70 years: NICE-compliers vs. usual care	1.25 [0.90–1.68]	1.09 [0.83–1.37]	1.04 [0.82–1.27]
Men aged >70 years: NICE-compliers vs. usual care	0.79 [0.53–1.07]	0.86 [0.66–1.08]	1.03 [0.86–1.19]
Women: NICE-compliers vs. usual care	0.81 [0.14–1.60]	0.95 [0.42–1.50]	1.04 [0.69–1.39]

* Bootstrap confidence interval.

### Men

Of the 112 patients in this study who had a rEVAR, 66 patients (59%) were men aged >70 years and 27 patients (24.1%) were men aged ≤70 years. Of the 186 patients who had a rOSR, 59 patients (31.7%) were men aged ≤70 years and 97 patients (52.2%) were men aged >70 years. Men aged ≤70 years showed slightly better survival under usual care than under compliance to NICE, 79% survival at 30 days, 66.8% at one year and 60.2% at 5 years vs. 73.7% at 30 days, 63.8% at one year and 58.8% at 5 years, respectively Figure 2C. Men aged >70 years had better estimated survival under compliance to NICE in the early phase (up to 2 years) of follow-up: usual care 63.7% survival at 30 days, 51% at one year, 35.3% at 5 years vs. NICE-compliance 71.3% at 30 days, 57.6% at one year and 33.7% at 5 years Figure 2D. The respective risk ratios are given in Table 3.

### Women

Overall 19 patients (17%) who had undergone rEVAR and 30 patients (16.1%) who had a rOSR were women. Women slightly benefitted from compliance to NICE in the early post-procedural phase: Under usual care survival probability was estimated as 70.7% at 30 days, 57.4% at one year and 37% at 5 years, compared to 76.2% at 30 days, 59.6% one year, 34.4% at 5 years under compliance to NICE Figure 2B.

### Hemodynamic stability—modified NICE criteria

**Figure 3:**
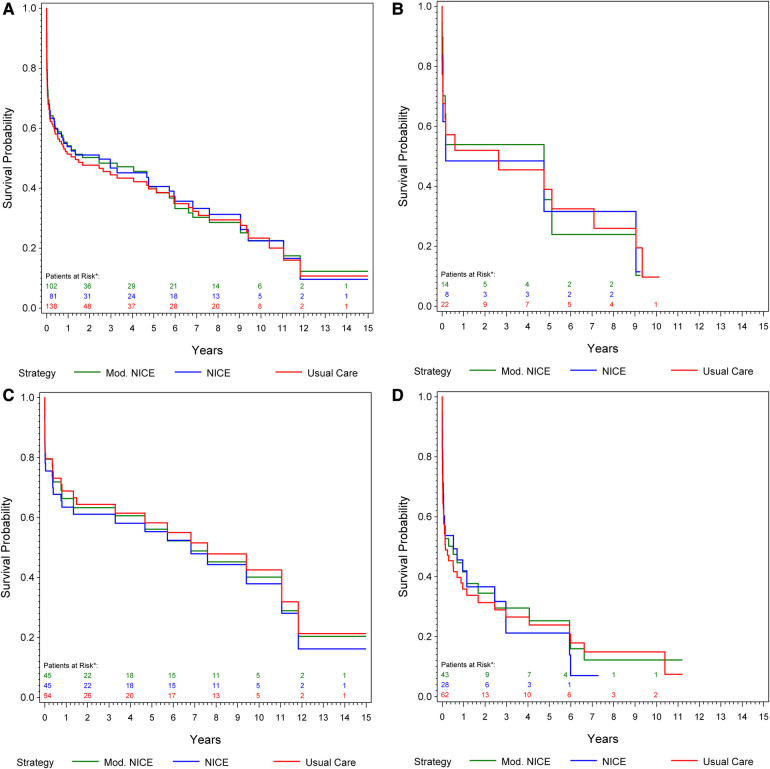
(**A**) Kaplan–meier curve: cumulative survival of patients from center 1 undergoing rOSR and rEVAR according to the NICE criteria, and to the modified NICE criteria, respectively, compared to the usual care cohort. (**B**) Kaplan–Meier curve of female patients: cumulative survival of patients from center 1 undergoing rOSR and rEVAR according to the NICE criteria, and to the modified NICE criteria, respectively, compared to the usual care cohort. (**C**) Kaplan–Meier curve of men aged ≤70 years: cumulative survival of patients from center 1 undergoing rOSR and rEVAR according to the NICE criteria, and to the modified NICE criteria, respectively, compared to the usual care cohort. (**D**) Kaplan–Meier curve of men aged >70 years: cumulative survival of patients from center 1 undergoing rOSR and rEVAR according to the NICE criteria, and to the modified NICE criteria, respectively, compared to the usual care cohort. *The actually observed numbers of patients at risk in the NICE-complier cohort, and the NICE-modified-complier cohort, respectively, are given. However, IPW-weighted observations were used for calculation of the Kaplan-Meier curve.

Data relating to patient hemodynamic stability was unavailable for 46 patients of center 1, and 1 patient of center 2 (15.8%). At presentation, 74.5% of patients undergoing rEVAR and 54.8% undergoing rOSR were deemed hemodynamically stable. The stability of the patients was defined according to the main clinical definition of blood pressure of at least 80 mmHg mean systolic on arrival (8). In a subgroup analysis of 138 patients (from center 2), we compared the estimated overall survival under usual care to that under compliance to mNICE (including hemodynamic stability) and NICE. Compliance to mNICE did marginally improve survival in contrast to compliance to NICE and usual care: Survival under mNICE was estimated as 69.4% at 30 days, 54.1% at one year and 40.4% at 5 years, compared to 68.1% survival at 30 days, 53.9% at 1 year and 40.6% at 5 years under NICE compliance, and 67.8% survival at 30 days, 51.3% at 1 year and 39.8% at 5 years under usual care Figure 3A and Table 4. Female patients seem to show a slight advantage under mNICE strategy in the early post-procedural phase, 70.2% survival at 30 days, 53.9% at 1 year, 35.6% at 5 years vs. NICE-strategy 61.6% survival at 30 days, 48.5% at one year, 31.6% at 5 years and usual care 67.6% survival at 30 days, 52.0% at 1 year, 39.0% at 5 years, respectively Figures 3B–D. The estimated risk ratios are given in Table 4.

**Table 4:** Subgroup analysis in 138 patients from center 2: risk ratios (RR) of mortality at 30 days, 1 year and 5 years: overall, in men aged ≤70 years, in men aged above 70 years and in women of all ages.

	Overall all-cause mortality		
	30 days RR [95% BCI*]	1 year RR [95% BCI*]	5 years RR [95% BCI*]
Overall: NICE-compliers vs. usual care	0.99 [0.73–1.29]	0.95 [0.76–1.14]	0.99 [0.83–1.15]
Overall: mNICE-compliers vs. usual care	0.95 [0.79–1.12]	0.94 [0.83–1.05]	0.99 [0.89–1.09]
Men aged ≤70 years: NICE-compliers vs. usual care	1.20 [0.91–1.56]	1.17 [0.98–1.42]	1.07 [0.91–1.26]
mNICE-compliers vs. usual care	1.01 [0.76–1.24]	1.08 [0.91–1.26]	1.05 [0.89–1.21]
Men aged >70 years: NICE-compliers vs. usual care	0.94 [0.57–1.34]	0.91 [0.66–1.16]	1.03 [0.83–1.24]
mNICE-compliers vs. usual care	0.97 [0.75–1.20]	0.91 [0.76–1.06]	0.98 [0.85–1.11]
Women: NICE-compliers vs. usual care	1.19 [0.00–2.54]	1.07 [0.30–1.92]	1.12 [0.50–1.79]
mNICE-compliers vs. usual care	0.92 [0.22–1.58]	0.96 [0.46–1.43]	1.06 [0.58–1.50]

* Bootstrap confidence interval.

.

## Discussion

The novelty of our study lies in the emulation of a hypothetical target trial, which compares survival outcome of patients treated according to the NICE criteria to patients' outcome under usual care using a retrospective data set from two aortic centres. Propensity score analyses and inverse probability of treatment weights (IPW) were used to equalize differences in baseline characteristics between the total (usual care) cohort and the sub-cohort of NICE-compliers (Supplementary Table S1) (13).

The results of this study provides further evidence to support the NICE recommendation to manage younger men (≤70 years) with hemodynamic stability with rOSR. Importantly, we did observe a slight survival advantage for NICE compliers in men >70 years and women of all ages. The NICE-modified strategy marginally increased survival in contrast to NICE-compliers and usual care, especially in female patients in the early phase.

In the long-term the early survival advantage seen in the NICE-compliers compared to usual care was lost. The results of this study potentially provides further support regarding the recommendation of the UK NICE Guidelines. In addition, it might be further suggested that older patients >70 years of age benefit from rEVAR as per the NICE-recommendation.

We did observe a clear early difference in survival in men aged >70 years following NICE compliance compared to usual care, even though this advantage was lost after 2 years. Similarly, men aged up to 70 years following usual care had a better outcome than corresponding NICE-compliers.

Importantly, long-term (5 year) survival in our cohort was poor irrespectivelly of treatment modality. The results of the IMPROVE trial demonstrated poor post-operative mortality in patients undergoing rEVAR and rOSR (14), but survival in our real-world observational cohort was even worse. Understanding the factors that contribute to these poor long-term outcomes represent an important area for future study. In addition, a non-inferiority meta-analysis of 3 RCTs, 21 observational studies and 8 registries, also showed rOSR to be non-inferior to rEVAR (15). However, sub-analysis of the data, with the data from RCTs only, showed a benefit favoring rEVAR compared to rOSR (15). This was supported by the conclusions of a recent Cochrane review emphasizing strong associations between rEVAR and improved post-operative outcomes, potentially advocating the adoption of a rEVAR first policy in patients presenting with rAAA (16–18). In line with the NICE-guidelines, we did observe survival advantage for NICE compliers in overall, men >70 years and women of all ages, which might possibly contribute further to the evidence of the current state of knowledge, favoring rEVAR first policy in ruptured abdominal aortic aneurysm.

It is important to appreciate the challenges when comparing rEVAR and rOSR retrospectively in a “real-world” setting such as this in comparison to a pragmatic RCT such as IMPROVE. Badgers et al. in a recent review came to the conclusion, that the paucity of data from prospective RCTs for the comparison of rEVAR and rOSR has made it difficult to decide which treatment modality is superior (17). Admittedly, there are several limitations that need to be acknowledged. A potential, drawback of the present study is its retrospective design. Nevertheless, retrospective data analysiscan add to the debate around the treatment decisions in rAAA and the potential need for further randomized studies whilst also identifying new areas for further exploration such as the factors driving poor long-term outcomes in this patient group. The combination of several new statistical methods to maximize the informative value of the study, potential modelling of physiological, anatomical and technical co-variates in relation to treatment modalities and outcome measures may help facilitate better patient selection as demonstrated previously (19). The RCT studies assume that patients may have been suitable for both OSR and EVAR, however this is an invalid assumption with rAAAs. Published NICE guidelines for AAA suggest patient hemodynamic stability and expert/resource availability should be the primary determinant of whether rOSR should be immediately attempted or patient assessed for suitability for rEVAR. However, in hemodynamically stable patients, current NICE guidelines recommend that younger men should be offered rOSR, whereas older men and women of any age should be offered rEVAR. The results of the present study provides further support for this strategy. However, we did not observe any significant differences in outcome in older patients between usual care and NICE compliance in long-term. Further studies are required in this area to build the evidence base that this is indeed the optimal approach for patients.

Furthermore, long-term analysis of AAA studies now advocate elective open surgical repair (OSR) in patients presenting an AAA predominantly due to improved long-term survival and cost effectiveness (7). Furthermore, OSR for elective AAA has been associated with reduced post-operative costs due to the need for actively surveillance, complications and subsequently re-intervention (7). Our study is in line with the concept that rOSR in men presenting with a rAAA ≤70 years is a reasonable approach, but it is important to be mindful that this is not a randomized study and therefore it is at risk of confounding bias. It is established that successful rOSR produces postoperative quality of life equivocal to the normal population (20, 21). Our study is limited by the modest sample size, especially in subgroup analysis and restricted to two geographical settings in Europe. On the other hand, the results of this study matches the evidence obtained from previously conducted RCT (22, 23). Furthermore, the prevalence of rAAA in most vascular centers throughout Europe is low compared to elective AAA intervention which is represented by the duration of data collection at both centers included in this study. As with all retrospective patient cohorts the possibility of residual confounding which cannot be factored in the analysis must be considered.

## Conclusion

Our evaluation of UK NICE Guidelines in ruptured infra-renal abdominal aortic aneurysms by emulating a hypothetical target trial provides further evidence recommending to manage younger men with hemodynamic stability with rOSR and older men, women and those with haemodynamic instability with rEVAR. Long term survival was poor and the early survival advantage of this strategy appeared lost over time. Application of the NICE Guidelines should be further evaluated in everyday work practice and the long-term outcomes of this patient group investigated further.

## Data Availability

The original contributions presented in the study are included in the article/**Supplementary Material**, further inquiries can be directed to the corresponding author.
