# Supplementary material for: Evaluation of national institute for health and care excellence guidance for ruptured abdominal aortic aneurysms by emulating a hypothetical target trial
Source: Front Cardiovasc Med. 2023 Jul 28;10:1219744. doi: 10.3389/fcvm.2023.1219744 (PMC10419256; doi:10.3389/fcvm.2023.1219744)
Supplement: Supplementary file 1 [file Table1.docx]

Supplementary Material

**Evaluation of National Institute for Health and Care Excellence (NICE) Guidance for Ruptured Abdominal Aortic Aneurysms (RAAA) by Emulating a Hypothetical Target Trial**

**Wolf Eilenberg^1^, Mohammed A Waduud^2,3^, Henry Davies^2,3^, Marc A Bailey^2,3^, D Julian A Scott ^2,3^, Florian Wolf^4^, Anna Sotir^1^, Sebastian Lakowitsch^1^, Alexandra Kaider^5^, Georg Heinze^5^, Christine Brostjan^1^, Christoph M. Domenig^1^, Christoph Neumayer^1*^**

^1^ Department of General Surgery, Division of Vascular Surgery, Medical University of Vienna, Austria.

^2^ Leeds Institute of Cardiovascular and Metabolic Medicine, University of Leeds, Leeds, United Kingdom.

^3^ Leeds Vascular Institute, Leeds General Infirmary, Leeds, United Kingdom.

^4^ Department of Biomedical Imaging and Image Guided Therapy: Division of Cardiovascular and Interventional Radiology, Medical University of Vienna, Austria

^5^Center for Medical Statistics, Informatics, and Intelligent Systems, Section for Clinical Biometrics, Medical University of Vienna, Austria

***Correspondence:** Christoph Neumayer, MD

E-mail: [christoph.neumayer@meduniwien.ac.at](mailto:christoph.neumayer@meduniwien.ac.at)

Department of General Surgery, Division of Vascular Surgery, Medical University of Vienna Waehringer Guertel 18-20, A-1090, Vienna, Austria

**Supplemental Table S1**: Results of Propensity Score Analyses:
Standardized Mean Differences (SMD) (NICE compliers – NICE non-compliers)

| Variable | unweighted SMD | | IPW-weighted SMD | |
| --- | --- | --- | --- | --- |
|  | Center 1 | Center 2 | Center 1 | Center 2 |
| Age | 0.140 | -0.376 | 0.009 | 0.039 |
| Gender | 0.011 | 0.397 | 0.002 | 0.009 |
| Diabetes Mellitus | 0.023 | -0.087 | 0.013 | -0.023 |
| Chronic Lung Disease | -0.112 | -0.026 | -0.002 | 0.036 |
| Chronic Kidney Disease | 0.009 | 0.024 | 0.003 | -0.009 |
| Stability |  | -0.334 |  | -0.010 |
